# Beneficial effects of Se/Zn co‐supplementation on body weight and adipose tissue inflammation in high‐fat diet‐induced obese rats

**DOI:** 10.1002/fsn3.2203

**Published:** 2021-05-06

**Authors:** Motahareh Hasani, Atoosa Saidpour, Pardis Irandoost, Fereshteh Golab, Maryam Khazdouz, Mostafa Qorbani, Fahimeh Agh, Ali Mohammad Sharifi, Mohammadreza Vafa

**Affiliations:** ^1^ Department of Nutrition School of Public Health Iran University of Medical Sciences Tehran Iran; ^2^ National Nutrition and Food Technology Research Institute (Department) Faculty of Nutrition Sciences and Food Technology Shahid Beheshti University of Medical Sciences Tehran Iran; ^3^ Cellular and Molecular Research Center Iran University of Medical Science Tehran Iran; ^4^ Non‐communicable Diseases Research Center Alborz University of Medical Sciences Karaj Iran; ^5^ Chronic Diseases Research Center, Endocrinology and Metabolism Population Sciences Institute Tehran University of Medical Sciences Tehran Iran; ^6^ Stem cell and regenerative Medicine research center and department of pharmacology School of Medicine Iran University of Medical Sciences Tehran Iran

**Keywords:** high‐fat diet, inflammations, obesity, oxidative stresses

## Abstract

This research investigated the effect of co‐supplementation of selenium with zinc on weight control and the inflammatory and oxidative status in relation to obesity. Male Wistar rats (*N* = 32) were randomly divided into four groups after induction of obesity model: 1) “Zn” was supplemented with zinc sulfate (15 mg/kg BW), 2) “Se” supplemented with selenium as sodium selenate (0.5 mg/kg BW), 3) “Zn + Se” which received Zn (15 mg/kg BW) + Se (0.5 mg/kg BW), and 4) “HFD” as the control group. The intervention was done for eight weeks. At the end of treatment, serum and tissue level of Zn, Se, SOD, GSH‐Px, MDA, leptin, TNF‐α, and IL‐6 was evaluated. Weight and food intake were significantly reduced in the Se group(*p* < .001), while in the Zn group, weight gain due to obesity was prevented compared to the control group (*p* = .48). There was a significant and stronger increase in SOD, GSH‐Px levels and a remarkable decrease in MDA, leptin, TNF‐α, and IL‐6 in the group receiving the combination of two supplements than either alone(*p* < .001). Leptin had a positive correlation with inflammatory factors and lipid peroxidation marker and showed an inverse relationship with Zn and Se levels and anti‐oxidative enzymes(*p* < .05). The analysis showed the mediating role of leptin in the effects of zinc. Co‐supplementation of selenium and zinc may have a synergistic effect in reduction of oxidative and inflammatory markers. Regarding the effect of zinc on inflammatory factors and lipid peroxidation, leptin can play a mediating role.

## Graphical abstract

This article revealed that co‐supplementation of selenium and zinc may have a synergistic effect in the reduction of oxidative and inflammatory markers. Regarding the effect of zinc on inflammatory factors and lipid peroxidation, leptin can play a mediating role.

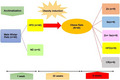

## 1 INTRODUCTION

The rise in the prevalence of obesity has become a global concern with consequences such as increased mortality and morbidity, so it has become a major threat for public health worldwide (Hosseini et al., 2017). Obesity is a complex, multistage, and preventable disease that brings with it a chronic inflammatory condition and oxidative status associated with comorbidities such as type 2 diabetes, cardiovascular disease, hypertension, and chronic kidney disease (Kotsis et al., 2010). In pathological obesity, adipose tissue undergoes a process called “adipose tissue regeneration,” which is characterized by increased levels of immune cell infiltration including interleukin‐6 (IL‐6), tumor necrosis factor‐alpha (TNF‐α), and leptin and eventually causes chronic low‐grade inflammation (Sun et al., 2011). On the other hand, recent evidence indicates that excess body fat may enhance oxidative stress, and concentrations of circulating antioxidants are inversely related to body mass index (BMI). In contrast, lower than normal levels of some antioxidants may be linked with increased fat deposition in the body so oxidative stress is associated with the spread of obesity (Hosseini et al., 2017; Wang et al., 2010). Thus, antioxidants can protect cells from oxidative stress and its consequences by trapping free radicals.

Zinc (Zn) is the most common microelements in the human body and plays an important role in growth and development (Hara et al., 2017). This metal participates in the regulation of chronic inflammatory status by reducing inflammatory cytokines. It also reduces oxidative stress by participating in the synthesis of antioxidant enzymes such as superoxide dismutase and glutathione peroxidase. Therefore, it acts as a catalyst for enzymes and is involved in lipid, carbohydrate, and protein metabolism (Ahn et al., 2014; Motamed et al., 2013). Epidemiological studies show that low Zn intake and low Zn blood concentrations are associated with an increased prevalence of obesity (Atkinson et al., 1978; Hosseini et al., 2017). Moreover, obese subjects frequently have hypozincemia and hyperzincuria (Chen et al., 1997, 1998).

Also, zinc may play a regulatory role in serum leptin concentration and appetite control (Mantzoros et al., 1998). Leptin is an adiposity signal that is secreted by the adipocytes in proportion to the amount of body fat. In healthy people, it regulates the energy balance by increasing energy expenditure and reducing energy intake (Chan et al., 2003). In diet‐induced obesity, leptin levels increase owing to leptin resistance, a condition deriving from activation of an inflammatory pathway, and systemic oxidative stress, and involved in disorders related to obesity and inflammation (Leon‐Cabrera et al., 2013). The results of the Baltaci and Mogulkoc study suggest that there is a complex association between zinc and leptin in terms of food intake regulation and cellular immune response, and zinc may play an important role in regulating the effects of the leptin hormone (Baltaci & Mogulkoc, 2012).

Besides zinc, selenium (Se) as an antioxidant plays a key role in the metabolism of thyroid hormones, redox reactions, reproduction, and immune function in the human body (Rayman, 2000). Selenium has beneficial effects on antioxidant, cancer prevention, diabetes, and metabolic syndrome (Chen et al., 2015; Volp et al., 2010). According to cross‐sectional studies, obesity is associated with low serum selenium levels and low intake of this nutrient (Kimmons et al., 2006; Wang et al., 2016). Several studies found blood concentrations of selenium (Se) inversely correlated with obesity, making Se deficiency a possible marker of adiposity (Bjørklund & Chirumbolo, 2017; Donma & Donma, 2016).

Taken together, a survey of the literature suggests a potential and promising role for Se or Zn supplementation in obesity and obesity‐related complications. Based on our knowledge, up to now, there has not been a report on the effect of combined selenium and zinc intervention on obesity caused by high‐fat diet, so we have hypothesized that supplementation of selenium with zinc, as a potential nutraceutical, may reduce weight and alleviate the inflammatory and oxidative status in relation to obesity. Furthermore, we aimed to examine whether Se and Zn could exert some of their effects through leptin increments.

## 2 MATERIALS & METHODS

### 2.1 Animals and treatment

A total of 40 male Wistar rats which were obtained from the Royan Institute (Tehran, Iran) at 4 weeks of age and weight of 50–70 g were housed separately in stainless steel cages in a well‐ventilated room maintained at 25 ± 2^°C^, with 12‐hr light/12‐hr dark cycles and 50%–55% relative humidity in the animal care facility at the National Nutrition and Food Technology Research Institute. Animals were given free access to a standard diet of commercial rat chow and tap water for 1 week. All animal experimental procedures were performed according to National Institutes of Health (NIH) guidelines for the care and use of laboratory animals. This study was approved by the Ethics Committee of the Iran University of Medical Science [IR.IUMS.REC.1397.577].

Following one‐week acclimatization, rats were randomly divided into high‐fat (*n* = 32) and normal (*n* = 8) groups. The standard diet given to the normal group had a caloric content of 3.5 kcal/g (65% carbohydrates, 10% lipids, and 25% proteins). The rats in the high‐fat group received HFD which had a caloric content of 5.6 kcal/g (20% carbohydrates, 20% proteins, and 60% lipids). HFD was prepared by Royan Institute for Biotechnology. The composition of HFD and ND is shown in Table 1.

**TABLE 1:** Composition of the experimental diets (per 1 kg)

Diets nutrients	ND	HFD
Casein (g/kg)	140	180
Cornstarch (g/kg)	630	50
Sucrose (g/kg)	100	10
Soy oil (g/kg)	40	40
SFA1 (g/kg)	‐	483
Fiber (g/kg)	50	50
Calcium	9.5	5.93
Phosphorus	6.5	4.06
As carbohydrate (%)	65	20
As fat (%)	10	60
As protein (%)	25	20
Energy (kcal/g)	3.6	5.6

Abbreviations: HFD, high‐fat diet; ND, normal diet.

**FIGURE 1:**
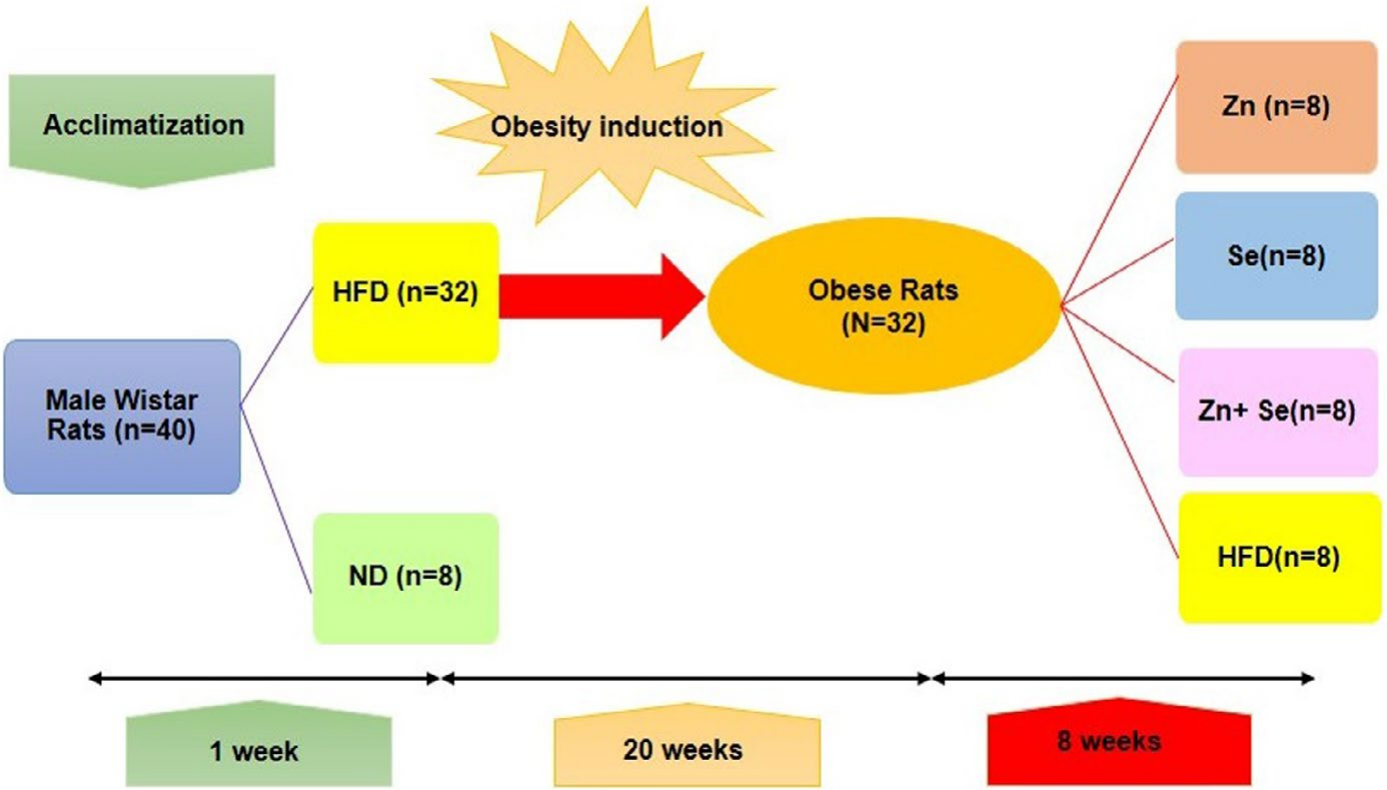
Scheme of the study protocol

When the weight of rats in the high‐fat group was significantly higher than the normal group after 20 weeks (473.18 ± 35.49 g versus 393.25 ± 22.30 g; *p* <.05) about 25% more than the normal group, an obesity model was induced and the treatment began for eight weeks. At the end of the 20th week, rats were randomly divided into four groups: “ 1) Group Zn” was supplemented with zinc sulfate (15 mg/kg BW), 2) “ group Se” was supplemented with selenium as sodium selenate ( 0.5 mg/kg BW), 3) “group Zn + Se” which received Zn (15 mg/kg BW) in combination with Se ( 0.5 mg/kg BW), and 4) “group HFD” without any intervention as the control group (Figure 1). The supplementation was done for eight weeks. Administered doses of Zn and Se were determined according to previous studies based on no observed adverse effects (Kumar et al., 2014; Mousavi et al., 2018; Wang et al., 2012; Zeng et al., 2012). HFD, supplemented with determined doses of Zn and Se, was weighed and given to rats every day at 9:00 a.m. The amount of food intake (the amount of food spilled the day before ‐ the amount left) was calculated and recorded daily, and the weight of the animals was measured and recorded weekly.

### 2.2 Sample collection

At the end of treatment, after 12 hr fasting, animals were anesthetized with 50 mg/kg of xylazine hydrochloride and 100 mg/kg of ketamine hydrochloride; then blood was collected via cardiac puncture. The plasma was immediately separated by low‐speed centrifugation at 2,500 × g for 15 min at 4°C and stored at −80^o^C. The inguinal WAT were removed, rinsed with phosphate‐buffered saline (PBS), and then tissue specimens were frozen in liquid nitrogen and stored at −80°C for posterior analysis.

### 2.3 Biochemical analysis

#### 2.3.1 Determination of zinc and selenium levels

Adipose tissue (150mg) was heated in silica crucibles using an oven furnace at 550^ºC^ for 24 hr, and the ash was taken up in hot hydrochloric acid (3 N) for Zn and Se analysis by atomic absorption spectrophotometer. Serum Zn level was determined by atomic absorption spectrophotometer.

#### 2.3.2 Determination of antioxidant enzymes activities

Measurement of SOD and GPX activity: SOD and GPX activity in serum and adipose tissue homogenate was determined using Rat Immunoassay kits (Bioassay Technology Laboratory (BT Lab), Inc., Shanghai, China) according to the manufacturer's protocols, based on the ELISA technique.

#### 2.3.3 Measurement of Lipid peroxidation

The lipid peroxidation level was measured as malondialdehyde (MDA), which is the end product of lipid peroxidation reacting with thiobarbituric acid (TBA) as a TBA reactive substance (TBARS) to produce a red‐colored complex with a peak absorbance at 532 nm.

#### 2.3.4 Determination of leptin and inflammatory markers

Enzyme‐linked immunosorbent assay (ELISA) Kits were used to determine leptin (MyBioSource, Inc. San Diego, California, United States). The concentrations of serum TNF‐α and IL‐6 were measured using the ELISA technique with commercially available kits (CUSABIO, Inc., Houston, USA) according to the manufacturer's protocols.

### 2.4 Statistical analysis

All values are given as mean ± standard deviation (*SD*). Differences and significance between groups were evaluated using one‐way analysis of variance (ANOVA), followed by Tukey's post hoc test analysis for multiple comparisons. To evaluate and compare the effect size, the standardized mean difference and its confidence interval were calculated. A *t* test was performed to compare the difference between two groups, and a paired *t* test was used for testing differences within the group. Correlation analysis was performed using Pearson's correlation coefficient. General linear model (univariate) was used for the analysis of covariance. Baron and Kenny's method was used to investigate the mediating role of leptin in the effect of the intervention (zinc and selenium). The criteria for this method include the following: 1‐ The intervention has a significant effect on the considered factors, 2‐ the intervention has a significant effect on the mediator (leptin), 3‐ when leptin is considered as a covariate, the effect of the intervention on the assessed factors becomes weaker but remains significant (Baron & Kenny, 1986). All analyses were performed using STATA version 14. The MedCalc statistical software, version 18.0 (MedCalc Software Ltd, Ostend, Belgium), was used for drawing figures. Differences were considered to be significant at *p* <.05.

## 3 RESULTS

### 3.1 Body weight & dietary intake

**FIGURE 2:**
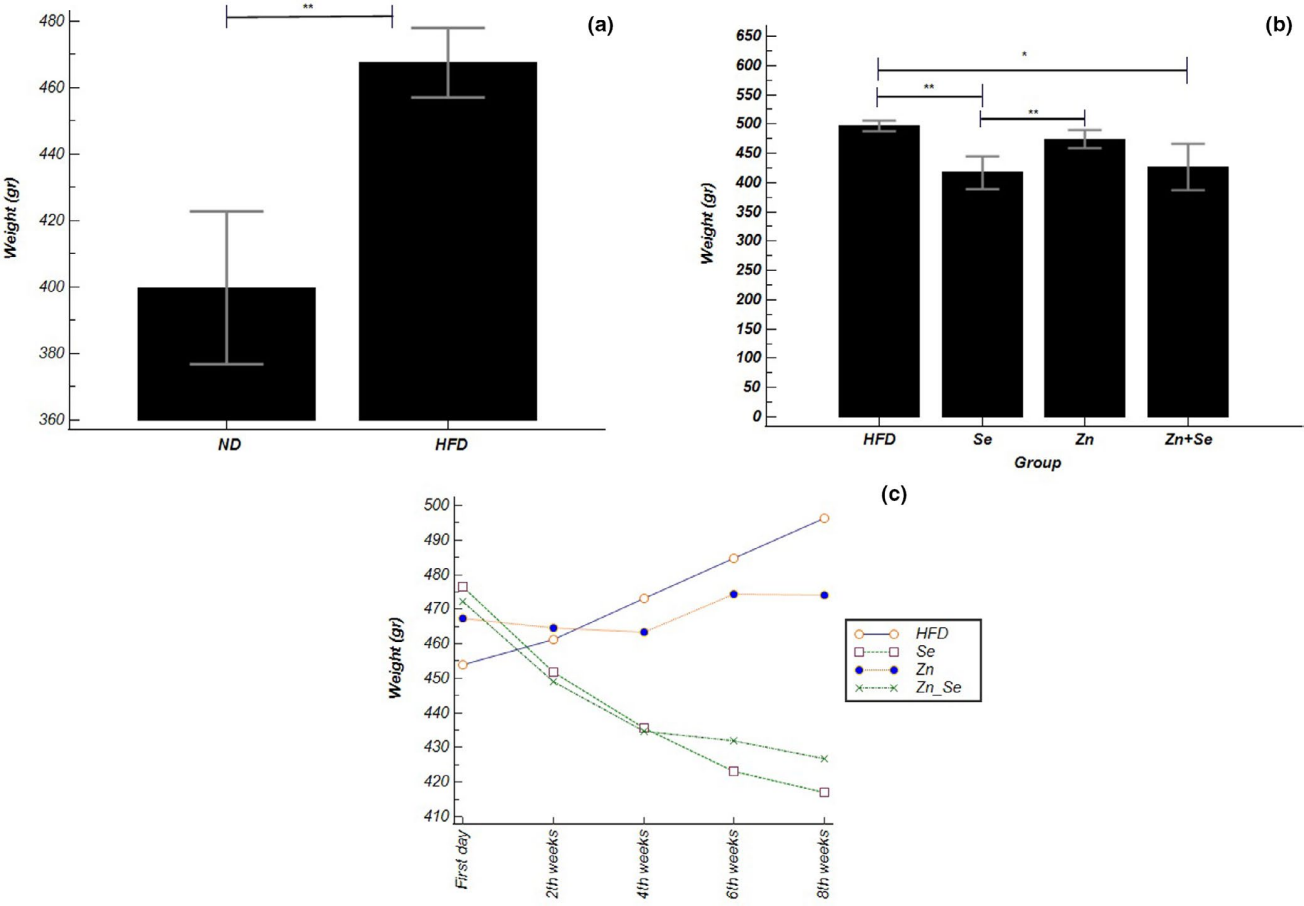
(a) Body weight in high‐fat diet (HFD) group versus normal diet (ND) groups; (b) comparison of body weight in the Zn (*n* = 8), Se (*n* = 8), Zn + Se (*n* = 8), and HFD groups; (c) comparison of body weight changes in identical times in interventional groups with repeated measure ANOVA. Data shown as mean ± *SD*; *p <.05, **p <.001

We did not lose any of animals during the study, and therefore, the interventions and analysis were performed on all rats until the end. The initial body weight of rats showed no significant difference between groups before the administration period. After 8 weeks of intervention, rats in the HFD group gained about 60(4.8) gr weight compared to the initial weight, while in the Zn group this amount increased by 6.67(8.1) gr which was approximately 12% of the weight gain observed in the HFD group, however, this difference between the two groups mentioned earlier was not statistically significant (*p* =0.488). The supplementation with Se and the combination of Zn and Se caused a significant decrease of about 53.40(12.3) gr and 45.62(11.8) gr, respectively, compared to the HFD group (*p* <.001). The results of weight changes in groups at the end of the intervention remained unchanged even after adjustment for baselines (Table 2). Weight loss due to Se administration was statistically substantial (*p* =.018) compared to changes due to Zn supplementation lonely but there was no considerable difference with the Se + Zn group (*p* =.992). Also, weight changes between the combination group and the Zn group were not significant (*p* =.075) (Figure 2).

Based on the results obtained from multiple comparisons of repeated measures ANOVA method, in the Se and the Se + Zn groups, both within‐group differences compared to the initial values and between‐group comparisons versus the control group at specific time points were significant (Se: p˂0.001, Se + Zn: p˂0.05). This was not the case with the zinc group (Figure 2).

The daily dietary intake of the rats has no meaningful differences among groups at the beginning and the end of intervention with multiple comparisons of the one‐way ANOVA method. However, considering the baseline intake as covariates of the final values and adjusting it, the daily food intake in the Se group showed a significant decrease compared to the HFD group (*p* =.001), Zn + Se group (p˂0.001), and Zn group (*p* =.001).

**TABLE 2:**
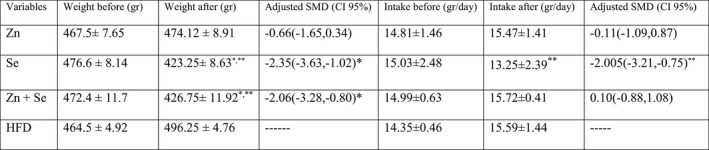
Comparison of weight and dietary intake in interventional groups before and the end of treatment

*Note:* Data are presented as the mean ± SEM. * between two intervention and control group (HFD) by one‐way ANOVA (Post hoc Tukey’s test); **Adjusted p‐value for baseline amounts.

Abbreviations: CRD, calorie restriction diet; HFD, high‐fat diet; SMD, standard mean differences with Cohen’s d.

### 3.2 Assessment of zinc and selenium content in serum and WAT

The obtained data demonstrated a significant increase in zinc concentration in serum and adipose tissue as a result of oral supplementation for 8 weeks in the Zn and Zn + Se groups comparing with HFD (p˂0.05). Serum level of selenium was increased in all three intervention groups that were significant compared to the control [Zn (*p* =.024), Se (p˂0.001), Zn + Se (*p* =.003)]. While this issue was not observed in adipose tissue and a significant increase occurred only in the group receiving selenium (p˂0.001) and the co‐supplementation group (*p* =.001).

### 3.3 The influence of zinc sulfate supplementation on selenium content and correlation between them

Along with increased zinc concentration, treatment with 15 mg/kg zinc sulfate significantly affected the serum level of selenium. Correlation analysis was performed to specify the association between Se and Zn in serum and tissue. A significant positive correlation between Se and Zn in serum and adipose tissue was observed in animals, respectively (r = 0.22, p‐value = 0.038, r = 0.51, p‐value = 0.0009).

### 3.4 Effects of Se and Zn supplementation on anti‐oxidative indices

**FIGURE 3:**
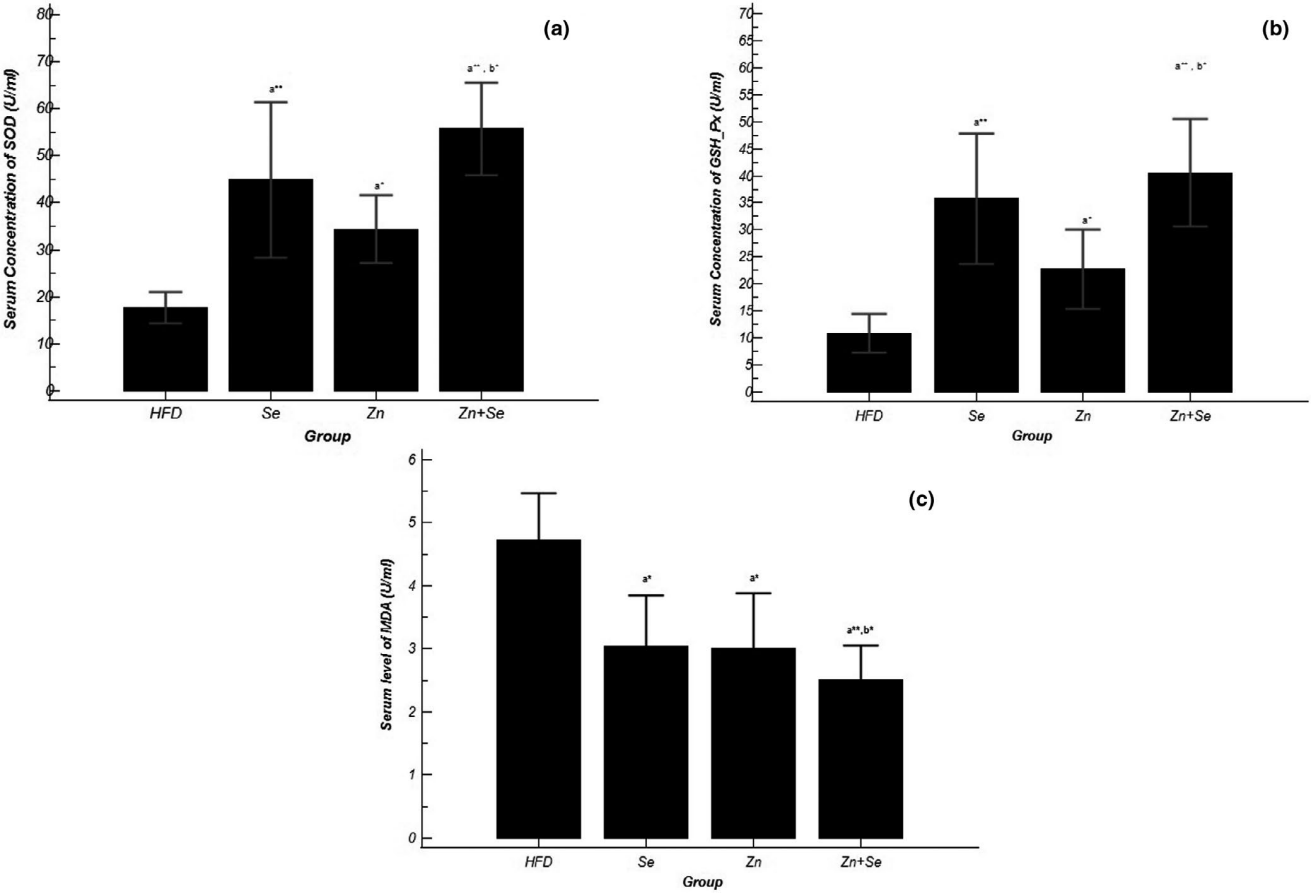
Comparison of (a) SOD concentration in serum, (b) GSH‐Px concentration in serum, (c) MDA concentration in serum in interventional groups; Data shown as mean ± *SD*; *p <.05, **p <.001

As shown in Table 3, the levels of serum antioxidant enzymes GSH‐Px and SOD were significantly increased in the Se and Zn + Se groups compared to HFD groups (*p* <.05). While in rats which received Zn, serum levels of SOD showed an increasing trend that was slightly different from the significant level (*p* =.068) and GSH‐Px did not show the difference between HFD groups. The level of SOD in adipose tissue showed a marked increase in all three intervention groups compared to the control group, while the tissue levels of GSH‐Px enzyme increased significantly only in Se and Zn + Se groups. Although an increase was observed in Zn group, it became marginally significant with a slight difference compared to the reference (*p* =.058). Our result showed that Se, Zn, and Zn + Se co‐supplementation significantly lowered the MDA levels in serum and adipose tissue compared with the HFD group (*p* <.05). However, the group receiving co‐administration of both the doses of Se and Zn showed a greater reduction in serum and tissue levels of MDA and remarkable increase in tissue levels of SOD that was considerable compared to groups which received Se or Zn supplementation alone (Figure 3).

**TABLE 3:** Comparison of anti‐oxidative and inflammatory indices in interventional groups

Variables	Zn	Se	Zn + Se	HFD
Leptin	4.70 ± 0.57	5.90 ± 0.83	5.21 ± 1.05	7.54 ± 1.29
P‐Value*	˂0.001	0.048	0.001	‐‐‐‐‐‐
SOD^S^	34.45 ± 8.71	44.89 ± 19.79	55.75 ± 11.90	17.73 ± 7.05
*p*‐Value*	.057	.001	˂.001	‐‐‐‐‐‐
*p*‐Value**	.129	.065	.086	
GPX^S^	22.68 ± 8.75	35.81 ± 14.47	40.54 ± 11.90	10.84 ± 4.23
*p*‐Value*	.224	˂.001	˂.001	‐‐‐‐‐
*p*‐Value**	.526	˂.001	.101	
SOD^T^	39.89 ± 7.28	51.42 ± 14.21	70.84 ± 17.88	18.40 ± 4.13
*p*‐Value*	.013	˂.001	˂.001	‐‐‐‐‐
*p*‐Value**	.171	˂.001	˂.001	
GPX^T^	20.97 ± 6.72	27.69 ± 9.37	28.66 ± 12.23	9.09 ± 5.10
*p*‐Value*	.058	.012	.007	‐‐‐‐‐
*p*‐Value**	.061	.003	.003	
MDA^S^	3.01 ± 1.04	3.03 ± 0.97	2.01 ± 0.65	4.73 ± 0.88
*p*‐Value*	.013	.015	.001	‐‐‐‐‐‐
*p*‐Value**	.021	˂.001	.003	
MDA^T^	159.07 ± 35.70	142.62 ± 37.77	121.36 ± 24.39	226.03 ± 52.30
*p*‐Value*	.023	.003	˂.001	‐‐‐‐‐‐
*p*‐Value**	.041	˂.001	.001	
TNF‐α^S^	64.41 ± 7.38	74.49 ± 5.90	52.60 ± 6.20	126.95 ± 8.20
*p*‐Value*	˂.001	˂.001	˂.001	‐‐‐‐‐‐
*p*‐Value**	.005	˂.001	.015	
IL−6^S^	76.59 ± 6.67	76.25 ± 12.01	59.15 ± 10.57	107.54 ± 16.53
*p*‐Value*	˂.001	˂.001	˂.001	‐‐‐‐‐‐
*p*‐Value**	.001	˂.001	.007	

Note

Data are shown as the mean ± *SD*; * P‐value is indicated versus HFD group by one‐way ANOVA(Post hoc Tukey's test); ** Adjusted for leptin. HFD: high‐fat diet. ^S^: measurement is indicated in serum level. ^T^: measurement is indicated in WAT.

Abbreviations: GPX, Glutathione peroxidase; IL‐6, Interleukin‐6; MDA, Malondialdehyde; SOD, Superoxide dismutase; TNF‐α, tumor necrosis factor‐α.

### 3.5 Effects of Se and Zn supplementation on leptin and anti‐inflammatory markers

**FIGURE 4:**
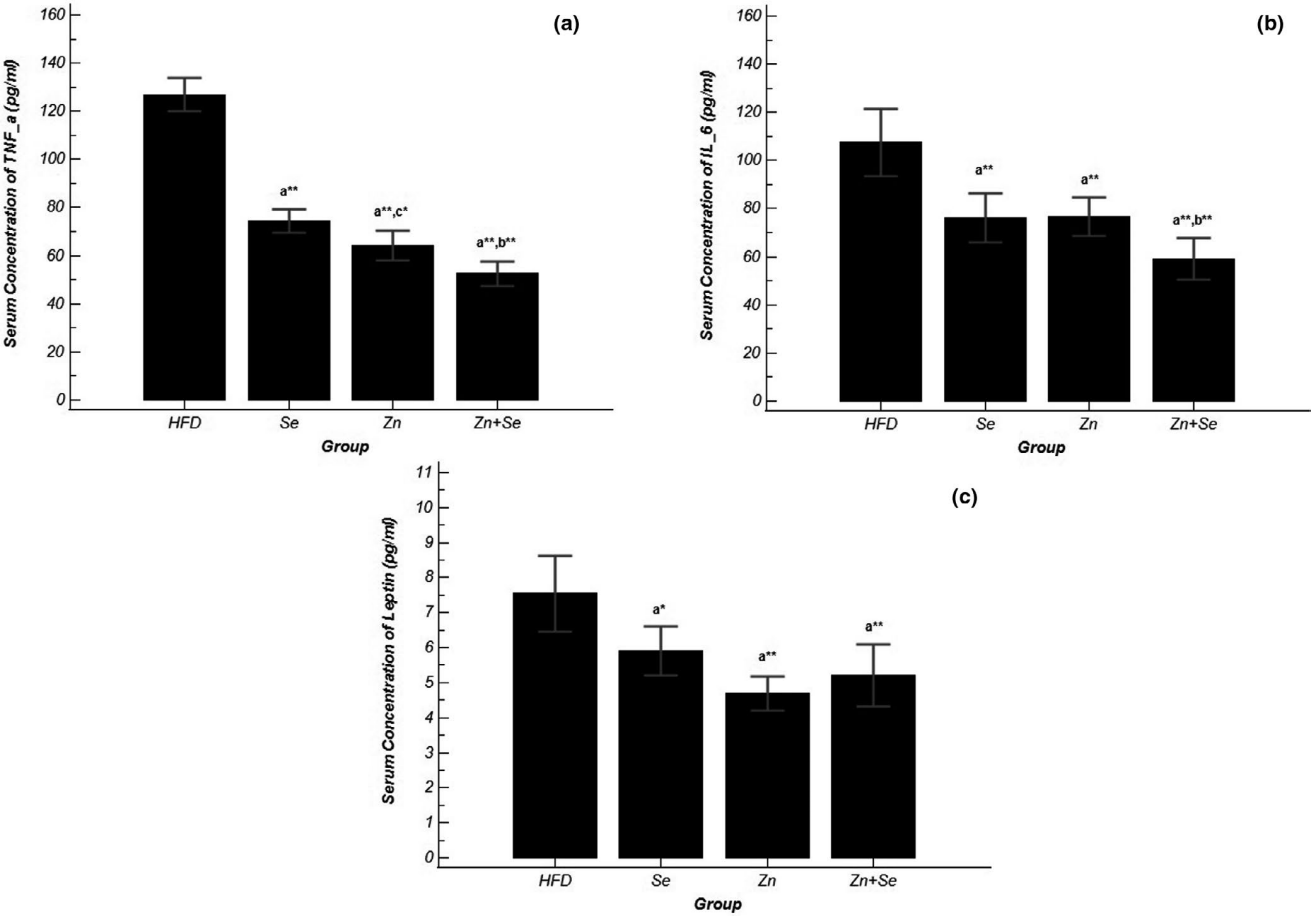
Comparison of (a) TNF‐α concentration in serum, (b) IL‐6 concentration in serum, (c) leptin concentration in serum in groups of treatment; Data shown as mean ± *SD*; *p <.05, **p <.001

As shown in Figure 4, the serum level of leptin was significantly reduced in all treatment groups; the greatest decrease was observed in Zn group (*p* ˂ .001). Serum concentration of inflammatory markers including TNF‐α and IL‐6 was significantly decreased in all intervention groups (*p* ˂ .001). The analysis showed that the reduction rate in the co‐administration group was substantially higher than the other two intervention groups (Table 3).

### 3.6 The role of leptin as a mediator

#### 3.6.1 Association of serum leptin level with selenium, zinc, anti‐oxidative, and inflammatory indices

The association between serum concentration of leptin and selenium, zinc, antioxidants, and inflammatory markers was evaluated based on Pearson's correlation test in obese rats (*n* = 32). As shown in Table 4, a negative correlation between serum leptin and Se, Zn, GSH‐Px, and SOD was observed. A significant positive correlation was found between the level of MDA, TNF‐α, IL‐6, and leptin levels.

**TABLE 4:** Correlation between serum leptin level and level of Zn, Se, anti‐oxidative, and inflammatory indices

variable	Zn	Se	GPX	SOD	MDA	TNF‐α	IL−6
*r*	−.36	−.31	−.487	−.484	.493	.695	.655
*p*‐Value	.021	.048	.006	.007	.20	˂.001	˂.001

Note:

Coefficients (*r*) and *p* values are calculated using Pearson's correlation analysis.

Abbreviations: GPX, Glutathione peroxidase; IL‐6, Interleukin‐6; MDA, Malondialdehyde; SOD, Superoxide dismutase; TNF‐α, tumor necrosis factor‐α.

#### 3.6.2 Assessing the effect of serum leptin as a mediator on improving the oxidative status and inflammatory parameters

**FIGURE 5:**
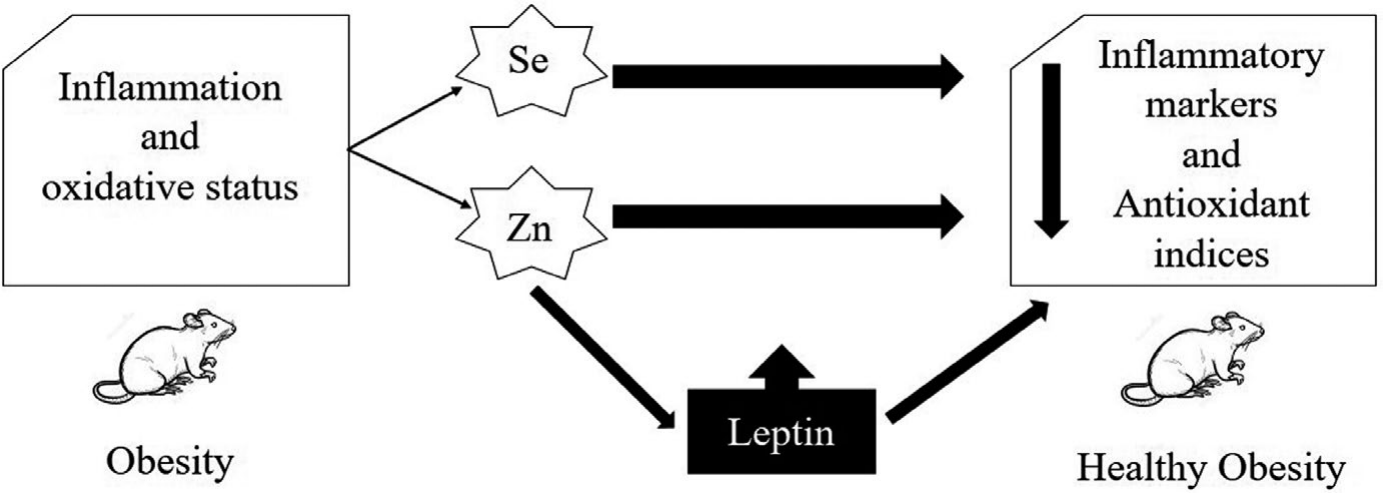
Diagram of leptin role as a mediator

Based on Baron and Kennyʼs method in defining a mediating factor, we first adjusted the effects of zinc, selenium, and co‐supplementation on anti‐oxidative and inflammatory factors for leptin (Figure 5). Then, we assessed the mediatory role of leptin on the anti‐oxidative and anti‐inflammatory effect of our interventions concerning all three conditions described in the method. In the present study, because all three conditions were met, leptin was identified as a mediator of Zn and Zn + Se co‐supplementation effects on inflammatory markers (TNF‐α and IL‐6) and MDA reduction. The results showed that the anti‐inflammatory and anti‐oxidative effects of zinc and the combination of zinc and selenium, although weakened, remained significant after leptin adjusting in comparison to the HFD group. These effects were not observed for the level of GSH‐Px and SOD. Leptin also did not meet the required conditions to consider as a mediator for the anti‐inflammatory and anti‐oxidative effects of selenium.

## 4 DISCUSSION

The present study evaluated the effects of zinc and selenium supplementation, alone and in combination, on obesity (weight, food intake, antioxidant status, and inflammatory markers). Also, for the first time, the synergistic effects of the combination of these two supplements on the complications associated with obesity were examined. On the other hand, the role of leptin as a mediator in the effects of zinc and selenium was investigated. The results of the present study showed that Se supplementation improves weight control in the obesity situation. In fact, rats in Se group lost about 11 percent of their weight before the intervention. The findings of the studies by Cavedon et al. (2020) and Zhang et al. (2018) were in line with ours. Also, in another experimental study, sodium selenite moderately attenuated the increased body weight of HFD‐fed mice (Nido et al., 2016). On the other hand, selenium intake reduced dietary intake in adjusted model in our research. However, there are disagreements in previous studies on the effects of selenium on weight and food intake (Falk et al., 2019; Mechlaoui et al., 2019; Nido et al., 2016). These contradictions flow from to the utilization of various sorts of selenium supplements or different doses in combination with other materials. This is incontrovertible fact that the consequences of selenium on weight are strongly dose‐dependent. Selenium in high doses has been shown to be ready to even cause weight gain (Zeng et al., 2012). However, the selenium‐induced weight loss observed in this study may be due to reduced dietary intake, as well as other factors like the effects of selenium on the genes involved in the mechanisms related to thermogenesis (Steinbrenner, 2013) or the effect on thyroid hormone activity (Guarino et al., 2018; Mahmoodianfard et al., 2015). Unfortunately, in the present study, the mentioned mechanisms have not been investigated and this is proposed for future studies. Unlike selenium, zinc supplementation showed an increasing trend in both weight and food intake but was not statistically significant. This results revealed that Zn supplementation ameliorated the increased body weight of HFD‐fed rats, although the difference was not considerable. There is controversy about the effect of zinc on weight and dietary intake in literature; however, several studies have been consistent with our results (Mousavi et al., 2018; Tallman & Taylor, 2003; Wang et al., 2012). Meanwhile, given the background on the association between zinc and weight, we measured the correlation between serum and tissue levels of zinc and animal weight and again found no significant relationship. Consistent with the newest meta‐analysis, it is still impossible to succeed in a conclusive results about the effect of zinc on weight (Abdollahi et al., 2020). About increasing food intake by consuming zinc, it can be considered that obesity is generally associated with zinc deficiency and its effects, one among which is anorexia, so with the administration of zinc and improvement of deficiency, anorexia disappears and food intake increases (Marreiro et al., 2006) that literature was consensus with our finding (Ghaemmaghami et al., 2010; Narjes Zare et al., 2020). Marked weight loss but less than the selenium group was seen in the combination group (9.5% of their weight before the intervention), and its food intake was not different from the control group. Perhaps the reason that the combination of the two supplements is less effective than selenium is the positive effects of zinc on weight and appetite in this research. So that, in the selenium group, significant weight loss was observed after the end of the first two weeks of the intervention and continued until the end, while in the combination group, a significant difference was observed from the middle of the intervention period.

In this research, a significant increase in serum and tissue levels of zinc and selenium was observed through supplementation. Also, based on the obtained results, it seems that zinc administration increases the level of Se due to the positive correlation shown with levels of this metal. In the study by Skalny et al. (Skalny et al., 2015), which was performed on rats, zinc supplementation increased selenium levels, which confirms the results of the current study. However, in Zn + Se group, although the selenium levels were increased significantly more than the control, but in compare to the Se and Zn groups, this did not happen.

In the same sense with the current findings, a wide variety of animal and human researches have shown an increase in SOD and GSH‐Px activity and decrease in MDA, TNF‐α, and IL‐6 with zinc and selenium intake (Asri‐Rezaei et al., 2015; El‐Demerdash & Nasr, 2014; Hasani et al., 2019; Nido et al., 2016; Prasad, 2014; Tomas‐Sanchez et al., 2018; Zhang et al., 2018). Of course, in our study, supplementation with zinc despite increasing the amount of GSH‐Px compared to the high‐fat group, but it was marginally significant in WAT that one of the reasons could be the low sample size, which affects the amount of P. value or power of statistical tests. To confirm this, we calculated the standardized mean differences, and at this stage, the effects of zinc on GSH‐Px became meaningful [SMD: 1.51, CI (0.36–2.62)]. However, the difference was insignificant in the serum concentration of GSH‐Px (β = 0.67). Furthermore, a remarkable increase in SOD activity and considerable decrease in MDA, TNF‐α, and IL‐6 were observed with Se and Zn co‐supplementation compared with individual administration. The synergistic effect of Se and Zn could be attributed to the improvement of anti‐oxidative and inflammatory indices. It should be noted that concomitant zinc and selenium deficiency is frequently observed in various pathological conditions (Çavdar et al., 2009; Cirino Ruocco et al., 2018; Khalili et al., 2008) as well as in healthy (Ghayour‐Mobarhan et al., 2005) and obese individuals (Brighenti et al., 1987). Based on these facts, it can be determined that there is a strong association between selenium and zinc homeostasis, which has been investigated and confirmed in various fundamental studies (Blessing et al., 2004; Feroci et al., 2005). Previous researches have determined the possibility of mutual influence and the potential for interactions between zinc and selenium in the case of supplementation (Guo et al., 2013). As in our study, we found that zinc intake alone can increase selenium levels. Putting these points together, it seems to be possible to emphasize the strong and two‐way association between Se and Zn as well as the synergistic effects of them in combination.

Our data indicate that Zn, Se, and co‐administration attenuated the elevation of leptin following HFD‐induced obesity. The strongest effect and decrease in leptin was seen in the Zn group compared to other intervention groups. Leptin resistance develops in obesity and causes hyperleptinemia. It is correlated with proinflammatory responses and increased levels of inflammatory markers observed in obesity. On the other hand, chronic inflammation caused by promoting proinflammatory cytokines such as IL‐6 and TNF‐a may interfere with leptin receptor signaling and impaired leptin function. Also, inflammatory cytokines (IL‐6 and TNF‐a) increase leptin production by adipose tissue. Increased leptin resistance is associated with high levels of free fatty acids may play a role in reducing fat oxidation in insulin‐sensitive organs and lead to fat accumulation (Izquierdo et al., 2019; Paz‐Filho et al., 2012). The latest systematic review on the role of leptin on inflammation and obesity is consistent with our findings (Pérez‐Pérez et al., 2020). According to previous in vitro study, NaSeO4 exposure can inhibit adipocyte differentiation, suppressing PPAR, C/EBP, and leptin gene expression in 3T3‐L1 preadipocytes (Kim & Kim, 2018). In a study by Cavedon et al., selenium supplementation for three months in obese individuals significantly reduced leptin levels that support our finding about selenium effect.

Based on literature, there is a complicate association between zinc and leptin in obesity conditions. The results of these researches revealed that zinc deficiency in obesity further increases the production and secretion of leptin; in contrast, zinc supplementation can reduce elevated levels of leptin and can be effective in improvement of the leptin resistance which occurs following obesity. Zinc can exert this reduction by interfering with the mechanisms involved in the production and secretion of leptin (Chen et al., 2000; Liu et al., 2013; Tallman & Taylor, 2003). Since our findings showed that the reduction of inflammatory markers and lipid peroxidation is related to a decrease in leptin, a hypothesis came to us that some of the anti‐inflammatory and anti‐oxidative effects of zinc may have been mediated by leptin. So, we performed the mediator analysis and it revealed that leptin acts as a mediator in reducing proinflammatory and oxidative markers. Consistent with results of the present study, in review article by Baltaci et al. revealed that there are complex and critical relationships between zinc and leptin in terms of both regulation food intake and cellular immune responses, but zinc may play an important role in regulating the effects of leptin (Baltaci & Mogulkoc, 2012). To the best of our knowledge, few studies have investigated the synergistic effect of Se and Zn co‐supplementation and on anti‐oxidative and anti‐inflammatory events and mediation role of leptin on the effects of zinc in obesity. There are variety of unknown mechanisms in this area. Therefore, more accurate evaluations are suggested to achieve a conclusive results.

## 5 CONCLUSIONS

In conclusion, the results of the present study revealed that selenium and zinc administration are beneficial in weight loss and inhibition of weight gain due to HFD‐induced obesity. Supplementation with the combination of selenium and zinc has stronger effects on reducing oxidative stress and chronic inflammation observed in obesity, which may suggest a synergistic effect of these two metals compared to receiving each individual. Also, leptin can act as a mediator in the effects of zinc on inflammatory factors and lipid peroxidation. However, more research is needed to find out precise mechanisms.

## CONFLICT OF INTEREST

The authors declare no conflict of interest. There is not any financial or personal relationships that might bias the work.

## ETHICAL STATEMENT

All animal experimental procedures were performed according to National Institutes of Health (NIH) guidelines for the care and use of laboratory animals. This study was approved by the Ethics Committee of the Iran University of Medical Science [IR.IUMS.REC.1397.577].
